# Age-stratified correlations between atopic dermatitis severity and biomarkers: hypercoagulability as a key indicator of severity in older adults

**DOI:** 10.3389/fmed.2026.1905694

**Published:** 2026-09-22

**Authors:** Yifang Zhang, Zhenghui Yang, Yingran Zhao, Hua Gu, Ying Tu

**Affiliations:** 1 Department of Dermatology and Venereology, First Afﬁliated Hospital of Kunming Medical University, Kunming, China; 2 Department of Dermatology and Venereology, Zhejiang Provincial People's Hospital Bijie Hospital, Bijie, China

**Keywords:** age stratification, atopic dermatitis (AD), biomarkers, disease severity, hypercoagulability, systemic inflammation

## Abstract

**Background:**

Atopic dermatitis (AD) is a chronic skin disorder with systemic inflammation. Previous studies have focused primarily on its allergic characteristics, whereas the accompanying systemic inflammation and potential hypercoagulable state, particularly across different age groups, have received insufficient attention.

**Objective:**

To investigate the correlations between AD severity and systemic inflammation, hypercoagulability, and allergic responses, and to evaluate the age-specific predictive value of these biomarkers.

**Methods:**

This cross-sectional study enrolled 181 patients with AD and 101 healthy controls. Disease severity was assessed using the Eczema Area and Severity Index (EASI). Blood samples were analyzed for allergic markers (total IgE and absolute eosinophil count), coagulation markers [D-dimer (D-D), fibrinogen degradation products, and D-dimer-to-fibrinogen ratio [DFR]], and systemic inflammation indices [systemic immune-inflammation index (SII), platelet-to-lymphocyte ratio (PLR), neutrophil-to-lymphocyte ratio, monocyte-to-lymphocyte ratio, and systemic inflammation response index]. Participants were stratified by age (<60 vs. ≥ 60 years). Group comparisons were performed using non-parametric tests, correlations were assessed using Spearman analysis, and predictive performance was evaluated using receiver operating characteristic curves.

**Results:**

All measured biomarkers were significantly higher in patients with AD than in controls (*P* < 0.05). Age stratification revealed age-dependent effects on several biomarkers. In the overall cohort, total IgE, SII, and PLR correlated positively with EASI scores (*r* = 0.184, 0.237, 0.266, respectively; *P* < 0.05), independent of age. In patients aged ≥60 years, absolute eosinophil count correlated with disease severity (*r* = 0.248, *P* < 0.05) and predicted moderate-to-severe AD [area under the curve (AUC) = 0.688]. Coagulation markers correlated with severity in both age groups but with distinct predictive patterns: DFR best predicted mild-to-moderate AD in patients <60 years (AUC = 0.723), whereas D-dimer best predicted moderate-to-severe AD in those ≥60 years (AUC = 0.805). Among the inflammation indices, SII and PLR predicted moderate-to-severe AD (AUC = 0.630 and 0.616, respectively).

**Conclusion:**

Patients with AD exhibit significant systemic inflammation, a subclinical hypercoagulable state, and enhanced allergic responses. Total IgE, SII, and PLR are reliable age-independent severity biomarkers. The diagnostic utility of coagulation markers and eosinophil counts is age-dependent. Based on these results, we suggest a practical, age-stratified clinical approach. In all patients with AD, total IgE, SII, and PLR were measured as core severity markers. In patients aged <60, DFR (AUC = 0.723) was used to distinguish mild-to-moderate disease. For those aged ≥60, D-dimer (AUC = 0.805) and the absolute eosinophil count were prioritized (AUC = 0.688) to predict moderate-to-severe AD. Monitoring systemic inflammation and coagulation is clinically relevant, particularly in elderly patients with moderate-to-severe AD.

## 1 Introduction

Atopic dermatitis (AD) is a chronic, relapsing inflammatory skin disease affecting individuals of all ages and characterized by intense pruritus and substantial impairment of quality of life (1, 2). Advances in research have increasingly recognized AD not only as a skin disorder but also as a systemic inflammatory disease. Over the past three decades, the global prevalence of AD has markedly increased. In China, the reported prevalence increased from 0.69% in 1998 to 12.94% in 2014 (3), and AD currently represents the leading contributor to the disease burden among non-fatal skin conditions (4).

The pathophysiology of AD is complex, involving dysregulation of both adaptive and innate immunity, with a dominant role for type 2 helper T (Th2) cell-mediated inflammation. Key Th2 cytokines, interleukin-4 (IL-4) and interleukin-13 (5, 6), drive immunoglobulin E (IgE) production (7), promote eosinophilic inflammation (8, 9), and downregulate epidermal barrier proteins such as filaggrin (10). This inflammatory cascade perpetuates the itch-scratch cycle and compromises the skin barrier function. Critically, the inflammatory response is not confined to the skin; activated immune cells and circulating cytokines induce a systemic inflammatory state involving multiple pathways, including Th2, Th22, and Th17 pathways (11). This systemic involvement links AD to various comorbid conditions, such as asthma and allergic rhinitis, and may elevate the risk of cardiovascular disease, metabolic syndrome, and psychiatric disorders (12, 13).

Chronic inflammation is a well-established driver of hypercoagulability (14). Inflammatory cytokines activate the coagulation cascade, damage the vascular endothelium, and inhibit the fibrinolytic system, collectively promoting a prothrombotic state (15–17). Preliminary evidence in AD suggests elevated coagulation markers and an increased risk of venous thromboembolism (18–20). Thus, AD exerts profound effects on systemic immune balance, inflammatory tone, and vascular homeostasis, beyond the skin.

In clinical practice, serum total IgE and eosinophil counts are traditional core biomarkers for assessing the allergic phenotype of AD (21). However, their utility in reflecting the overall disease severity and systemic involvement is limited. Numerous studies have indicated that although these markers may be elevated in some patients (22–25), their correlation with clinical severity scores (e.g., Eczema Area and Severity Index [EASI]) is inconsistent, and their value in distinguishing disease activity or predicting treatment response remains constrained. This inconsistency suggests that the systemic impact of AD may not be fully captured by the allergic pathway markers alone.

In recent years, systemic inflammation indices derived from complete blood counts—such as the systemic immune-inflammation index (SII), neutrophil-to-lymphocyte ratio (NLR), and platelet-to-lymphocyte ratio (PLR)—have been widely investigated in oncology, cardiovascular diseases, and rheumatoid arthritis because of their accessibility and low cost (26–28). These indices serve as robust tools for assessing the systemic inflammatory burden and prognosis, reflecting the complex interplay among innate immunity, adaptive immunity, and platelet activation. Similarly, key markers of coagulation and fibrinolysis, such as D-dimer and fibrinogen degradation products (FDP), are often elevated in chronic inflammatory states, such as sepsis and autoimmune diseases (29), signifying a prothrombotic state and endothelial dysfunction, thereby bridging inflammation and thrombotic risk.

These newer indices have proven their worth in their respective corners, but they have never been combined to capture systemic inflammation and hypercoagulability in AD. Age sits squarely at the center of immune and coagulation homeostasis; however, age-stratified, multisystem biomarker analyses remain strikingly rare in the AD literature. Childhood AD is a distinct disease. The well-known Th2 dominance (30) tells only part of the story; the coagulation system is still maturing, and the pathophysiological machinery driving the disease differs fundamentally from that in adults. For these reasons, we confined our study to the adult span of 12–80 years and kept the 60-year threshold firmly in sight. Our working hypothesis was that age does more than tilt these biomarkers; it rewrites their predictive meanings. To test this idea, we compared allergic, inflammatory, and coagulation markers between patients with AD and healthy controls, examined how those markers tracked with EASI scores in younger (<60) versus older (≥60) adults, and evaluated the age-specific predictive power of each marker for different severity grades. The aim was practical from the start: to provide clinicians with an assessment framework that respects the patient's age.

## 2 Methods

### 2.1 Study design

This was a cross-sectional, observational study. We consecutively enrolled 181 patients diagnosed with atopic dermatitis at the Department of Dermatology, The First Affiliated Hospital of Kunming Medical University, between January 2020 and December 2024. A control group of 101 healthy volunteers who underwent routine health checkups or minor non-inflammatory surgeries during the same period was also recruited. The study protocol was approved by the Medical Ethics Committee of the First Affiliated Hospital of Kunming Medical University [Approval No.: (2025) Lun Shen L No. 4]. Written informed consent was obtained from all participants or their guardians.

The diagnosis of AD strictly adhered to the criteria outlined in the Chinese Guidelines for the Diagnosis and Treatment of Atopic Dermatitis (2020). Healthy controls had no history of AD or other type 2 inflammatory diseases (e.g., asthma and allergic rhinitis). All participants were aged between 12 and 80 years.

The exclusion criteria were as follows: (1) concurrent hematological diseases, eosinophilia, scabies, hereditary or immunobullous diseases, or other conditions potentially affecting the study biomarkers; (2) severe hepatic or renal insufficiency, or comorbid malignancy, syphilis, or HIV infection; (3) systemic use of corticosteroids or immunosuppressants within 2 months prior to enrollment; and (4) a recent history (within 3 months) of acute thromboembolic events, such as cerebral infarction, pulmonary embolism, or myocardial infarction.

For the healthy control group, in addition to the above exclusion criteria, we further excluded the following comorbidities and conditions that could potentially affect D-dimer and other coagulation marker levels: (1) history of cardiovascular diseases (including atrial fibrillation, heart failure, coronary artery disease, and peripheral arterial disease); (2) history of deep vein thrombosis or pulmonary embolism; (3) recent (within 3 months) surgery or trauma; (4) active infection or autoimmune diseases; (5) hepatic or renal insufficiency; and (6) current use of anticoagulant or antiplatelet agents.

### 2.2 Clinical assessment and grouping

Two experienced dermatologists independently assessed each patient with AD using the EASI. The EASI score was used to evaluate erythema, edema/papulation, excoriation, and lichenification across four body regions (head/neck, trunk, upper limbs, and lower limbs), with a total score ranging from 0 to 72. Based on the score, patients were categorized into three severity groups: mild (EASI ≤ 7), moderate (7 < EASI ≤ 21), and severe (EASI > 21). To investigate the influence of age, and based on the previous literature indicating that 60 years is a significant threshold for age-related changes in coagulation markers such as D-dimer, all study subjects (including patients with AD and healthy controls) were stratified into two age groups: <60 and ≥60 years. We chose 60 years as the age cutoff for two main reasons. Landmark studies on elderly patients with AD have consistently defined “elderly” as 60 years and older, a convention that keeps our results directly comparable with existing literature (31, 32). A deeper justification comes from physiology: after age 60, immune function undergoes accelerated immunosenescence, skin barrier properties shift, and coagulation/fibrinolysis homeostasis changes measurably—the well-known age-related climb in D-dimer is one example (14). The 60-year threshold draws from precedent and biological evidence alike.

However, our age-stratification design warrants caution. By placing everyone under 60 years of age into a single group, we pooled adolescents (12–17 years) with young and middle-aged adults (18–59 years). This was a deliberate choice, based on a prior study showing that 60 marks a genuine inflection point for coagulation markers such as D-dimer. We aimed to contrast the profiles of elderly and non-elderly adults. However, this grouping should not be read as treating adolescents and younger adults as a uniform population, nor does it license extrapolation to prepubertal children, whose immune development, coagulation homeostasis, and AD immune phenotypes stand apart from the adult context entirely.

### 2.3 Laboratory measurements

Fasting venous blood samples were collected from all subjects in the morning. A complete blood count was performed using an automated hematology analyzer to obtain the absolute eosinophil count and percentage, platelet count, and absolute counts of neutrophils, lymphocytes, and monocytes. Coagulation tests (D-dimer, fibrinogen, and fibrinogen degradation products) were conducted using the clotting method on an automated coagulation analyzer. Serum total IgE levels were measured using a chemiluminescence immunoassay. All assays were performed in the central laboratory of the First Affiliated Hospital of Kunming Medical University following standardized operating procedures.

The following systemic inflammation indices were calculated based on the complete blood count results:

Systemic immune-inflammation index (SII) = Platelet count × Neutrophil count/Lymphocyte count
Systemic inflammation response index (SIRI) = Neutrophil count × Monocyte count/Lymphocyte count
Neutrophil-to-lymphocyte ratio (NLR) = Neutrophil count/Lymphocyte count
Platelet-to-lymphocyte ratio (PLR) = Platelet count/Lymphocyte count
Monocyte-to-lymphocyte ratio (MLR) = Monocyte count/Lymphocyte count

In addition, the D-dimer-to-fibrinogen ratio (DFR) was calculated as a composite indicator of coagulation status.

### 2.4 Statistical analysis

The sample size was estimated using G*Power 3.1 software. Given that the data may not follow a normal distribution, non-parametric tests will be used for group comparisons. To determine the required sample size, we referred to a two independent samples *t*-test for a power analysis. With a significance level of *α *= 0.05 (two-tailed), a statistical power of 1−*β*=0.80, and a medium effect size of *d* = 0.5 according to Cohen's criteria, the calculation indicated that at least 64 participants were required per group, totaling at least 128 participants for both groups. In this study, we enrolled 181 patients with AD and 101 healthy controls, which exceeded the minimum sample size requirement.

Data analysis was performed using IBM SPSS Statistics (version 29.0). The normality of continuous variables was assessed using the Shapiro–Wilk test. Normally distributed data are presented as mean ± standard deviation and compared between groups using one-way ANOVA; non-normally distributed data are presented as median and interquartile range and compared using non-parametric tests (Kruskal–Wallis H test or Mann–Whitney *U* test). Categorical variables are presented as frequencies and percentages, and comparisons were made using the chi-square test or Fisher's exact test. The correlation between biomarkers and EASI scores was analyzed using Spearman's rank correlation test. The predictive performance of each biomarker for disease severity was evaluated using receiver operating characteristic (ROC) curve analysis, calculating the area under the curve (AUC) with a 95% confidence interval. The optimal cutoff value, sensitivity, and specificity were determined using the Youden index. All statistical tests were two-sided, and a *P*-value of < 0.05 was considered statistically significant.

## 3 Results

### 3.1 Demographic and baseline characteristics

A total of 181 patients with AD and 101 healthy controls were enrolled. No statistically significant differences were observed between the two groups in terms of age or gender distribution (both *P* > 0.05), indicating comparability (Table 1). Given previous evidence suggesting that elderly patients with AD (aged ≥60 years) have lower peripheral blood eosinophil counts compared to pediatric and adult patients with AD (31), we further stratified both patients with AD and controls into two age groups (<60 and ≥60 years) to explore the potential influence of age on the study biomarkers. The distribution of patients with AD and controls within the <60 and ≥60 years subgroups showed no significant difference (*P* > 0.05, *χ*^2^=2.215).

**Table 1:** Comparison of demographic characteristics between patients with AD and healthy controls.

Characteristic	Patients with AD (*n* = 181)	Healthy controls (*n* = 101)	*P*-value
Age, years [median (IQR)]	58 (48, 68)	56 (38, 69)	0.282
Age, *n* (%)			0.847
<60 years	100 (60.6)	65 (39.4)	
≥60 years	81 (69.2)	36 (30.8)	
Gender, *n* (%)			0.107
Male	101 (55.8)	46 (45.5)	
Female	80 (44.2)	55 (54.5)	

The median EASI score was 15.1 (11.5, 22.45) for male patients and 13.5 (9.60, 18.90) for female patients, with no statistically significant difference (*P* = 0.061). The median ages for patients with mild, moderate, and severe AD were 56 (47.75, 65.25), 58 (47, 68), and 60 (54, 69) years, respectively, showing no significant difference among severity groups (*P* = 0.431). Furthermore, there was no significant difference in EASI scores between patients with AD aged <60 years [median 14.8 (9.925, 19.65)] and those aged ≥60 years [median 15.0 (11.55, 21.6)] (*P* = 0.338).

AD in older adults follows several distinct clinical trajectories. To capture this heterogeneity, we drew on published criteria (32) and divided our 81 patients (all ≥60 years) into three groups: late-onset AD (first symptoms at ≥60 years), adult-persistent AD (onset in adulthood and persisting into old age), and childhood-onset AD with late-life relapse (onset in childhood and relapse in old age). The cohort was dominated by late-onset disease (63 patients, 77.8%), followed by adult-persistent AD (18, 22.2%); no patient was in the childhood-onset category. We then compared the laboratory and clinical markers between the late-onset group (*n* = 63) and the adult-persistent group (*n* = 18). Inflammatory markers (SII, PLR, NLR, MLR, and SIRI), coagulation parameters (D-dimer, FDP, and DFR), allergic indices (total IgE and absolute eosinophil count), and EASI scores were statistically indistinguishable between the two groups (all *P* > 0.05). Regardless of whether AD first appeared in adulthood or later in life, the link between hypercoagulability and disease severity was firm in our elderly patients. This constancy points to a shared pathophysiology—above all, the interplay between systemic inflammation and a hypercoagulable state—which operates irrespective of when the disease begins.

### 3.2 Comparison of biomarkers between patients with AD and healthy controls

Compared to healthy controls, patients with AD exhibited a generalized elevation across all three categories of biomarkers: allergic, coagulation, and systemic inflammation. The median levels of absolute eosinophil count and percentage, D-dimer, FDP, DFR, SII, NLR, PLR, MLR, and SIRI were significantly higher in the AD group (all *P* < 0.01) (Table 2). The differences in coagulation and inflammation markers were particularly consistent and significant.

**Table 2:** Comparison of biomarker levels between patients with AD and healthy controls.

Biomarker	Healthy controls (*n* = 101)	Patients with AD (*n* = 181)	*Z*-value	*P*-value
Allergic/inflammatory markers				
Absolute EOS count (×10⁹/L)	0.08 (0.045,0.155)	0.32 (0.165,0.515)	−9.198	<0.001
EOS (%)	1.50 (0.75,2.25)	5.00 (2.60,8.60)	−9.343	<0.001
Total IgE (IU/mL)	Not tested	91.450 (28.225,247)	-	-
Coagulation/fibrinolysis markers				
D-dimer (mg/L)	0.22 (0.15,0.28)	0.29 (0.195,0.585)	−4.763	<0.001
FDP (mg/L)	1.20 (1.00,1.76)	1.9 (1.31,2.75)	−6.019	<0.001
DFR	0.073 (0.056,0.097)	0.110 (0.077,0.223)	−6.284	<0.001
Systemic inflammation indices				
SII	459.080 (313.247,611.396)	521.994 (355.734,829.622)	−2.788	0.005
NLR	2.043 (1.549,2.472)	2.220 (1.632,3.857)	−2.689	0.007
MLR	0.229 (0.175,0.292)	0.316 (0.224,0.434)	−6.049	<0.001
PLR	121.514 (100.371,159.460)	143.386 (115.209,194.523)	−3.527	<0.001
SIRI	0.743 (0.558,1.254)	1.125 (0.665,1.972)	−4.157	<0.001

Data are presented as median (IQR). EOS, eosinophils; FDP, fibrinogen degradation product; DFR, D-dimer-to-fibrinogen ratio; SII, systemic immune-inflammation index; PLR, platelet-to-lymphocyte ratio; NLR, neutrophil-to-lymphocyte ratio; MLR, monocyte-to-lymphocyte ratio; SIRI, systemic inflammatory response index; NA, not applicable.

### 3.3 Impact of age stratification on biomarker levels

Age-stratified analysis revealed that age is a key factor modulating the levels of multiple biomarkers in patients with AD and the magnitude of their difference from healthy controls.

First, for allergic markers, patients with AD had significantly higher absolute eosinophil counts and percentages than their age-matched healthy controls in both the <60 and ≥60 years strata (all *P* < 0.01). Notably, increasing age was associated with a significant increase in eosinophil levels among patients with AD: those aged ≥60 years had significantly higher absolute eosinophil counts and percentages than those aged <60 years (*P* < 0.01). This age-associated increasing trend was not observed in the healthy controls (*P* = 0.132).

Second, analysis of coagulation markers showed that regardless of age, patients with AD had significantly higher D-D, FDP, and DFR levels compared to age-matched healthy controls (all *P* < 0.001). Age itself was also a significant determinant of these markers. In both the general healthy population and among patients with AD, individuals aged ≥60 years had significantly higher D-D and DFR levels than those aged <60 years. Specifically, FDP levels showed a significant age-dependent increase in patients with AD, a phenomenon not present in healthy individuals.

Importantly, advancing age appears to have an additive effect on the elevation of certain biomarkers in AD. For example, eosinophil counts and D-D levels in patients with AD aged ≥60 years were not only higher than those in age-matched healthy individuals but also showed a more pronounced increase compared to patients with AD aged <60 years (Figure 1), suggesting that systemic inflammation and hypercoagulability may be more prominent in elderly patients with AD.

**Figure 1:**
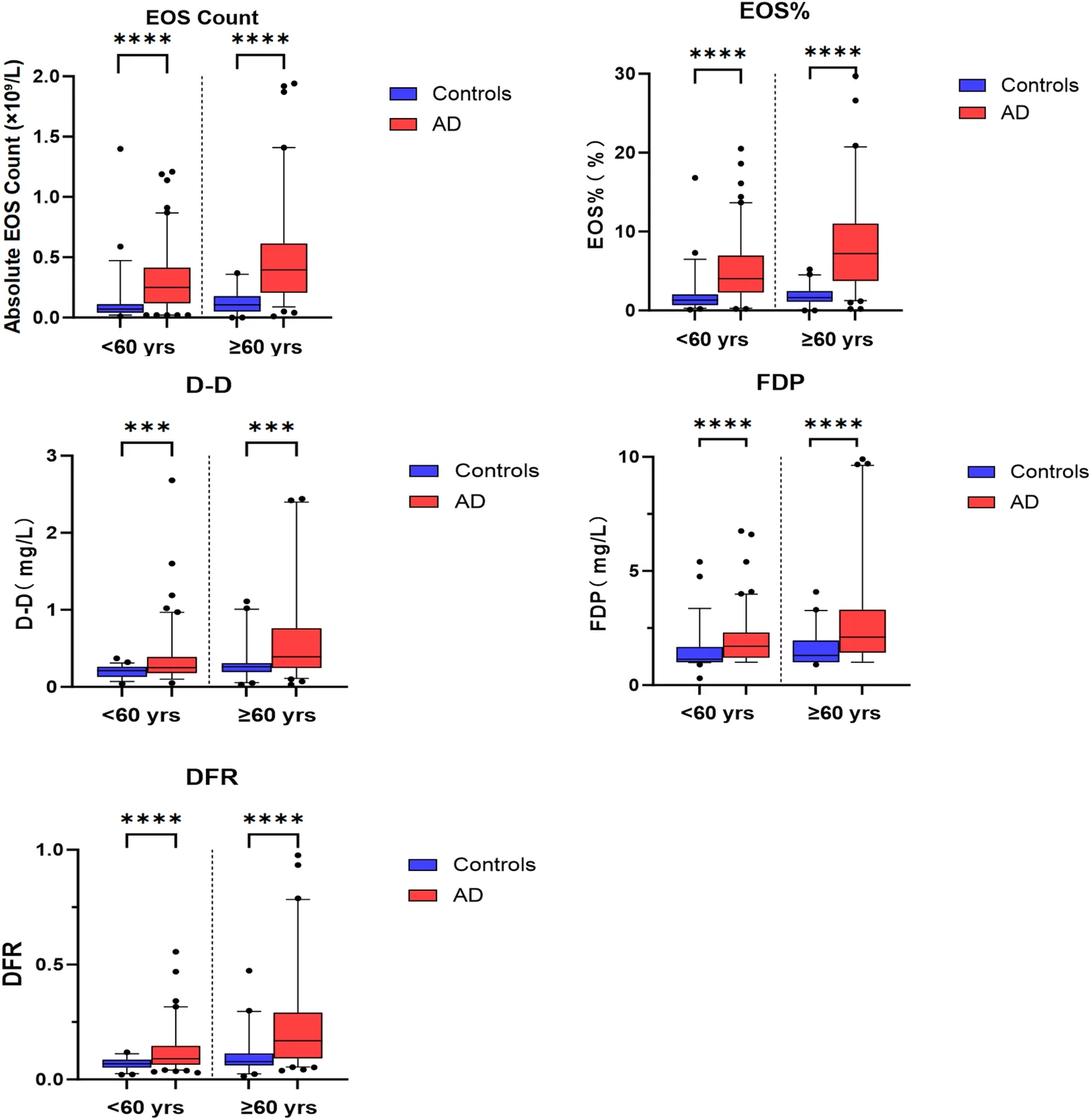
Age-stratified comparison of key biomarkers. *P < 0.05, **P < 0.01, ***P < 0.001, ****P < 0.0001; ns, not significant.

Finally, given that the baseline levels of systemic inflammation indices may be influenced by age, we performed age-stratified case–control comparisons. Among individuals aged <60 years, patients with AD had significantly higher NLR, MLR, and SIRI than healthy controls (all *P* < 0.05). In the ≥60 years stratum, patients with AD still exhibited significantly higher MLR and SIRI than controls (*P* < 0.05), but no significant difference was found in NLR between the two groups (*P* = 0.148). This indicates that after accounting for the effect of age on baseline levels, the elevation of MLR and SIRI in AD was consistent across age groups, while the elevation of NLR was primarily observed in younger patients.

### 3.4 Correlations between biomarkers and disease severity: overall and age-stratified analysis

The EASI score in patients with AD showed no significant association with gender (*P* = 0.061) or age (*P* = 0.338). Furthermore, the age distribution did not differ significantly among the different severity groups (mild, moderate, and severe) (*P* = 0.431).

In the overall AD cohort, total IgE levels and the systemic inflammation indices SII and PLR showed significant positive correlations with the EASI score. Coagulation markers (D-D, FDP, and DFR) exhibited a significant graded increase across severity groups (mild, moderate, and severe) and were significantly positively correlated with the EASI score (Table 3).

**Table 3:** Levels of key biomarkers across AD severity groups.

Biomarker	Mild AD (*n* = 22)	Moderate AD (*n* = 119)	Severe AD (*n* = 40)	Overall *P*-value
Total IgE (IU/mL)	105.90 (18.875,523.0)	57.20 (25.75,204.0)	203.50 (40.425,463.75)^ab^	0.008
D-D (mg/L)	0.23 (0.20,0.413)	0.30 (0.22,0.56)	0.675 (0.268,1.048)^ab^	<0.001
FDP(mg/L)	1.50 (1.00,1.805)	1.88 (1.4,2.6)^a^	2.85 (2.043,3.69)^ab^	<0.001
DFR	0.077 (0.066,0.144)	0.105 (0.075,0.185)	0.189 (0.097,0.366)^ab^	<0.001
SII	403.370 (315.665,767.744)	513.161 (347.674,823.590)	603.395 (475.082,1,053.245)^ab^	0.03
PLR	124.283 (104.810,174.102)	137.939 (112.153,190.873)	169.034 (123.748,229.641)^ab^	0.02

Data are presented as median (IQR).

a Significantly different from the mild AD group (*P* < 0.05).

b Significantly different from the moderate AD group (*P* < 0.05).

Overall correlation analysis confirmed that among all enrolled patients with AD, total IgE, SII, and PLR were significantly positively correlated with the EASI score (total IgE: *r* = 0.184, *P* = 0.014; SII: *r* = 0.237, *P* = 0.002; PLR: *r* = 0.266, *P* < 0.001). Coagulation markers also demonstrated strong correlations with disease severity, showing a significant graded increase across severity groups (all *P* < 0.001) and significant positive correlations with the EASI score.

Age-stratified analysis further revealed differences in the association patterns between the biomarker categories and disease severity.

Universal Correlation of Coagulation Markers: D-D, FDP, and DFR showed significant positive correlations with the EASI score in both patients aged <60 years and ≥60 years (Figure 2). In the <60 years group, the Spearman's rank correlation test confirmed positive correlations between the EASI score and plasma D-D level (*r* = 0.286, *P* < 0.01), FDP level (*r* = 0.279, *P* < 0.01), and DFR level (*r* = 0.326, *P* < 0.01). Similarly, in the ≥60 years group, the EASI score was positively correlated with the D-D (*r* = 0.359, *P* < 0.01), FDP (*r* = 0.311, *P* = 0.005), and DFR levels (*r* = 0.300, *P* = 0.006). The levels of these markers were significantly higher in patients with severe AD than in those with mild or moderate disease.

**Figure 2:**
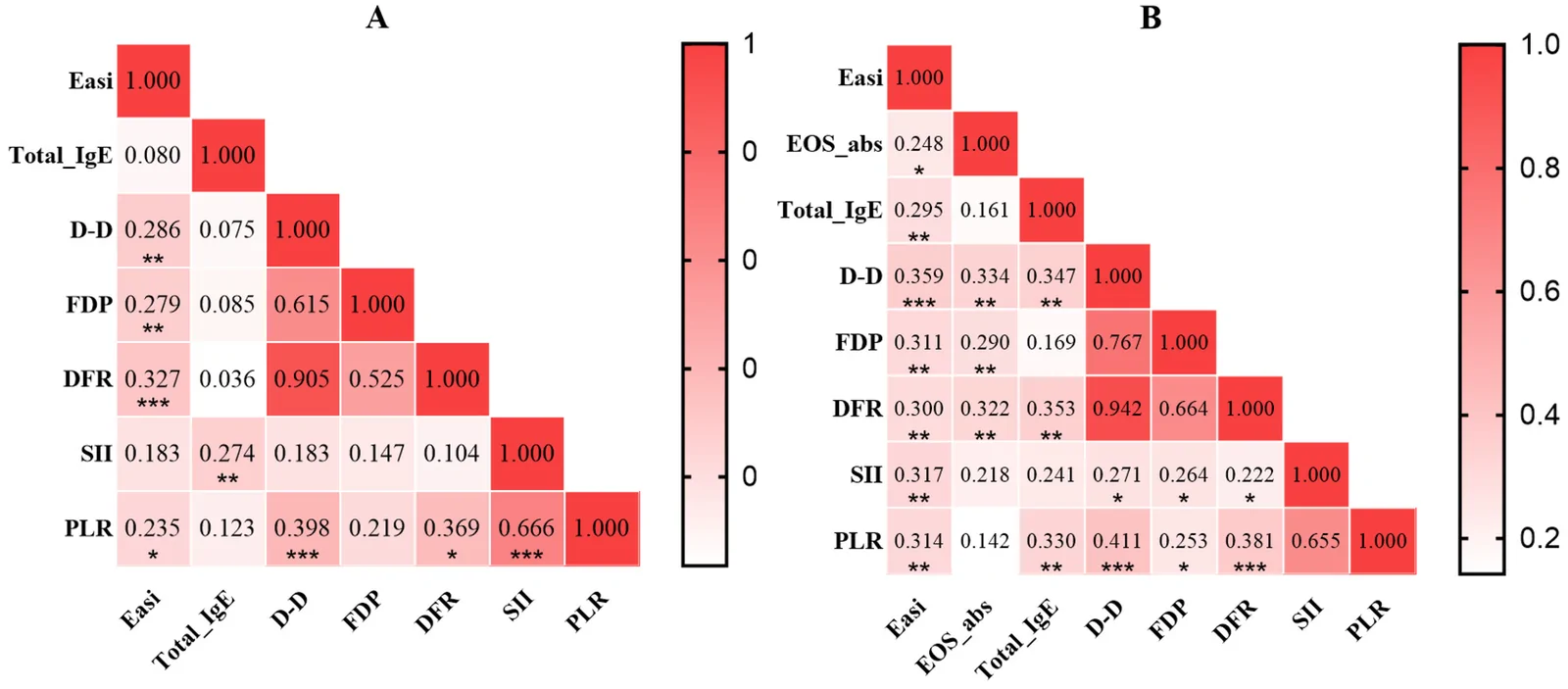
Spearman correlation heatmap of biomarkers with EASI score in patients with AD aged <60 years (**A**, *n* = 100) and ≥60 years (**B**, *n* = 81). Red indicates positive correlation and blue indicates negative correlation. *P < 0.05, **P < 0.01, and ***P < 0.001.

Age specificity of allergic markers: The correlation between total IgE and the EASI score observed in the overall analysis did not reach statistical significance in between-group comparisons within either age stratum after stratification (*P* = 0.24). The association of eosinophil count showed a clear age restriction: a significant positive correlation between absolute eosinophil count and EASI score was found only in patients with AD aged ≥60 years (*r* = 0.248, *P* = 0.026, Figure 2B), with severe patients having significantly higher eosinophil levels than those with mild or moderate disease (*P* < 0.05). No significant association was found between eosinophil count and disease severity in patients aged <60 years.

Variability of Inflammation Indices: The correlations between SII and PLR with severity were established in the overall analysis. However, other inflammation indices (NLR, MLR, and SIRI), although elevated in patients with AD compared to controls in some age strata, showed no significant differences among the different severity groups within the AD patient population (all *P* > 0.05).

In summary, coagulation markers demonstrated the most robust correlation with AD severity, independent of age. Total IgE and the composite inflammation indices, SII and PLR, were important severity-related markers. The eosinophil count as a marker of disease activity showed significant age dependence, being primarily applicable to elderly patients with AD.

### 3.5 Predictive value of biomarkers for disease severity

To evaluate the clinical utility of the identified biomarkers, we analyzed their diagnostic performance in distinguishing different AD severity grades using ROC curve analysis (Figures 3–5).

**Figure 3:**
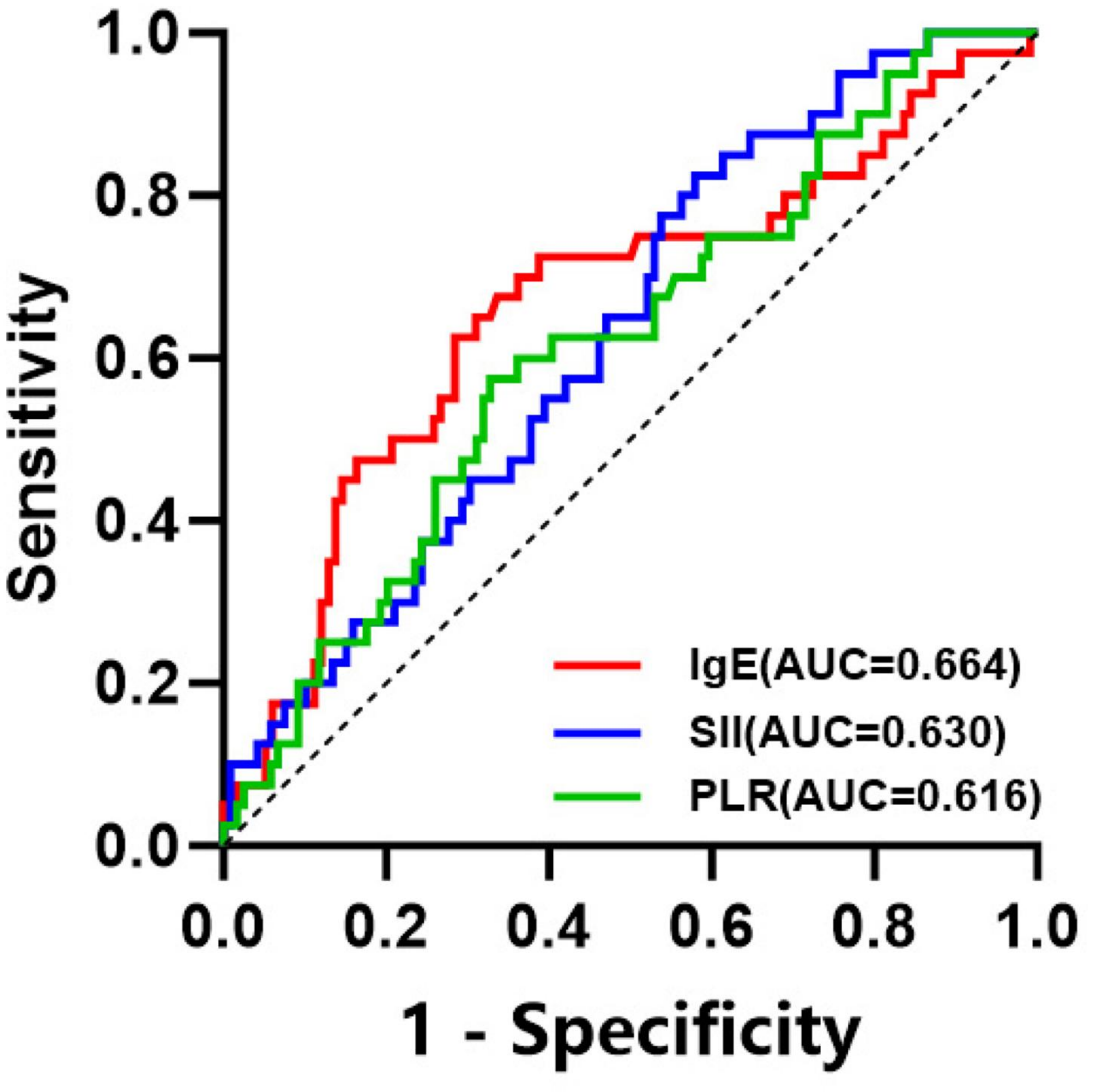
Predictive performance of systemic inflammation indices (SII and PLR) and total IgE levels for moderate-to-severe AD.

**Figure 4:**
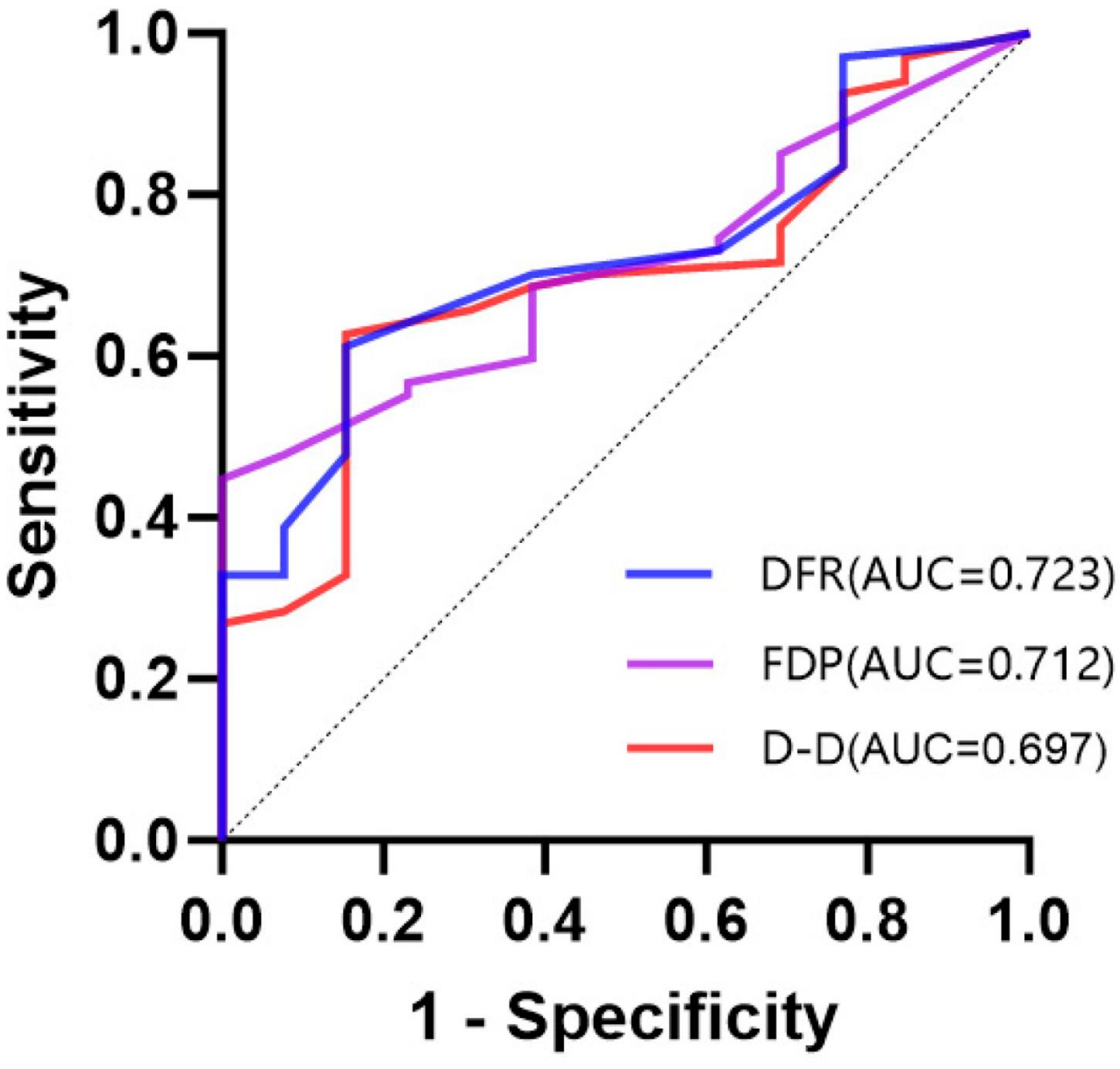
Predictive performance of coagulation markers for mild-to-moderate AD in patients aged <60 years.

**Figure 5:**
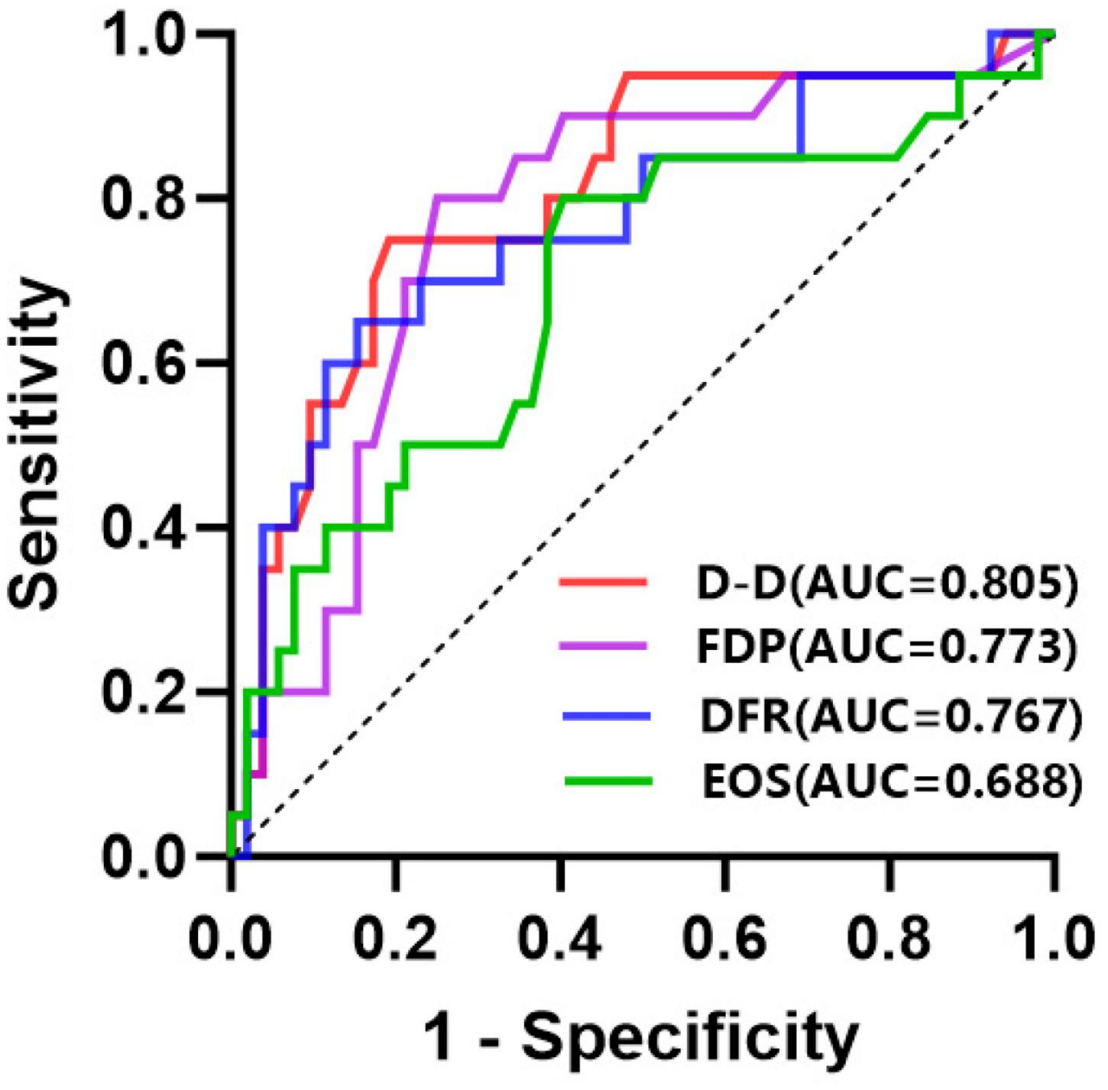
Predictive performance of coagulation markers and eosinophil count for moderate-to-severe AD in patients aged ≥60 years.

In the overall cohort, total IgE showed moderate predictive value for distinguishing moderate-to-severe AD (AUC=0.664, 95% CI: 0.56–0.768). The SII and PLR also demonstrated significant, albeit slightly lower, predictive ability for moderate-to-severe AD (AUC=0.630 and 0.616, respectively); however, they were ineffective in distinguishing mild-to-moderate AD (Table 4, Figure 3).

**Table 4:** Predictive performance of overall biomarkers for moderate-to-severe atopic dermatitis.

Biomarker	AUC	Optimal cutoff	Sensitivity (%)	Specificity (%)	95% CI	Youden index	* P*-value
Total IgE	0.664	152.0 IU/mL	0.625	0.716	0.561–0.768	0.341	0.002
SII	0.630	430.5	0.825	0.420	0.536–0.723	0.245	0.014
PLR	0.616	161.8	0.575	0.672	0.517–0.715	0.247	0.028

**Table 5:** Age-stratified predictive performance of coagulation markers for AD severity.

Prediction target	Age group (years)	Marker	AUC	Optimal cutoff	Sensitivity (%)	Specificity (%)	95% CI	* P*-value
Mild-to-moderate	<60	D-D	0.697	0.235	0.627	0.846	0.559–0.835	0.025
		FDP	0.712	1.805	0.448	1	0.590–0.835	0.016
		DFR	0.723	0.085	0.612	0.846	0.595–0.851	0.011
	≥60	All markers	Not significant					
Moderate-to-severe AD	<60	All markers	Not significant					
	≥60	D-D	0.805	0.750	0.75	0.808	0.688–0.922	<0.001
		FDP	0.773	3.050	0.80	0.750	0.651–0.894	<0.001
		DFR	0.767	0.277	0.65	0.846	0.636–0.899	<0.001
		EOS	0.688	0.395	0.80	0.596	0.543–0.833	0.014

Age-stratified analysis revealed more targeted prediction models. Among patients with AD aged <60 years, coagulation markers were of significant value for identifying early disease progression (i.e., distinguishing mild-to-moderate AD). DFR showed the best predictive performance (AUC = 0.723, 95% CI: 0.595–0.851), with a specificity of 84.6% at an optimal cutoff value of 0.085 (Table 5, Figure 4).

Conversely, among elderly patients with AD aged ≥60 years, the predictive value of biomarkers was primarily evident in identifying severe disease. D-D was the strongest predictor for distinguishing moderate-to-severe AD (AUC=0.805, 95% CI: 0.688–0.922), with good sensitivity (75%) and specificity (80.8%). In addition, the absolute eosinophil count in this age group showed potential for predicting moderate-to-severe AD (AUC = 0.688; Table 5, Figure 5).

In summary, total IgE, SII, and PLR can serve as auxiliary indicators for assessing the overall severity of AD. In contrast, coagulation markers (DFR and D-D) and eosinophil counts provide age-specific predictive information, aiding in the identification of early disease progression in younger patients and severe disease states in elderly patients.

## 4 Discussion

In recent years, the understanding of the age spectrum of AD has undergone a significant shift. Traditionally considered a disease of childhood, AD with first presentation in older adults is often misdiagnosed as other eczematous disorders. Recent studies have shown that the prevalence of AD in the elderly (aged ≥60 years) has been gradually increasing. Elderly patients with AD can be classified into three types based on the age of onset: AD persisting from childhood or adolescence with relapse in the elderly, AD persisting and/or relapsing from adulthood, and AD with onset in the elderly (aged >60 years) (32). Among these, the late-onset type predominates, accounting for approximately 47.3% of elderly patients with AD (31). This conceptual update has led to an increasing recognition of elderly patients with AD as a distinct entity, whose pathogenesis, immune phenotype, and systemic impact may differ fundamentally from those of younger adult AD.

D-dimer levels were substantially higher in patients with AD than in age-matched healthy controls, and this elevation was observed across both age strata—the <60 group and the ≥60 group (Table 2). When set against our strict exclusion criteria, this age-stratified result strongly suggests that AD-driven systemic inflammation raises D-dimer levels independently of the chronological age. In other words, the signal tracks with the disease itself, not with aging or the common comorbidities that so often blur the picture.

Unlike the immune profile of younger adult AD, that of elderly patients with AD is not dominated solely by type 2 inflammation. Wang et al. (31) reported that elderly patients with AD had significantly lower peripheral blood eosinophil counts than children and younger adults, along with elevated levels of IL-4, IL-17A, IL-22, and other cytokines, indicating a mixed Th2/Th17/Th22 inflammatory pattern. In contrast to the above findings, the present study revealed that elderly patients with AD (aged ≥60 years) had significantly higher absolute eosinophil counts than younger patients (aged <60 years) (*P* < 0.01), and eosinophil counts were positively correlated with disease severity (*r* = 0.248, *P* = 0.026). Several factors may account for these discrepancies. First, the median EASI score of the elderly patients in our cohort was 15.0, which was higher than that reported by Wang et al., and eosinophil counts are known to correlate with disease activity. Second, differences in demographic characteristics, environmental exposures, and genetic backgrounds across regions may contribute to observed heterogeneity. Third, a considerable proportion (47.3%) of elderly patients in our study had late-onset AD (first onset after age 60 years), which may exhibit distinct immune phenotypes compared with those with AD persisting from an earlier age (10). Nevertheless, both our study and that of Wang et al. support the notion that elderly patients with AD display a more complex immune profile. This “inflammaging” phenomenon predisposes elderly patients with AD to systemic presentations. Our study found significantly elevated coagulation markers (D-dimer and FDP) and eosinophil counts in elderly patients with AD, which were closely correlated with disease severity, further supporting the systemic inflammatory signature of elderly patients with AD. Therefore, beyond skin manifestations, clinical assessment should pay greater attention to cardiovascular disease risk in elderly patients with AD, particularly those with a hypercoagulable state. Previous studies have linked chronic inflammatory diseases, such as AD, to an increased risk of atherosclerosis and venous thromboembolism (18, 19, 33, 34), and our data suggest that hypercoagulability may be a key pathological link.

The immunosenescence and inflammaging processes described above extend well beyond shifts in cytokine profiles and a revved-up coagulation system. They also cause deeper immune dysregulation and platelet dysfunction. Within the immune compartment, an age-driven tilt in the balance between regulatory T cells (Tregs) and Th17 cells likely sits at the heart of the broken immune tolerance and relentless inflammatory amplification that mark elderly patients with AD (35, 36). Schmitt et al. showed that, even at rest, older individuals carry higher numbers of Th17 cells and fewer Tregs—an imbalance that skews the immune response away from regulation and toward inflammation (35). As Treg function wanes, the suppression of effector T cells slackens (37); meanwhile, overactive Th17 cells pull neutrophils into the tissue and stoke local inflammation. These twin disruptions are well-positioned to fuel the mixed inflammatory pattern characteristic of elderly patients with AD (36). Platelets also bear the imprint of aging. Older platelets display greater basal activation, reflected in higher P-selectin expression and increased shedding of platelet microparticles (38, 39). Davizon-Castillo and colleagues took this further, demonstrating that age-related inflammation—especially elevated TNF-α—directly triggers platelet hyperreactivity and drives thrombus formation (38, 40). When AD-driven systemic inflammation is layered on top of age-related platelet hyperreactivity, the coagulation cascade ignites at a lower threshold. This mechanism likely explains why D-dimer levels in our elderly patients tracked disease severity more tightly (*r* = 0.359, *P* < 0.01). Clinically, this means that elderly patients with AD contend with more than the burden of skin disease; they also face a heightened thrombotic risk, powered jointly by immunosenescence and platelet activation (36). Consequently, managing moderate-to-severe AD in elderly patients requires more than controlling skin inflammation. Coagulation markers, such as D-dimer, require serial monitoring, and a multidisciplinary strategy should be adopted when cardiovascular risk factors are present.

Aging and inflammation feed into each other. As years accumulate, the immune system drifts into a state of chronic, low-grade activation—inflammaging—and this persistent inflammatory tone, in turn, accelerates immunosenescence. These two factors create a self-reinforcing loop (14, 16). In this context, chronic inflammation in AD may induce or exacerbate hypercoagulability by activating the vascular endothelium, upregulating tissue factor expression, and inhibiting the fibrinolytic system (15, 17). The significant positive correlation between D-dimer levels and EASI scores (AUC = 0.805) observed in elderly patients with AD in our study supports this mechanistic hypothesis. Based on these findings, we speculate that adjunctive therapies aimed at promoting blood circulation and resolving stasis may confer additional benefits in elderly patients with AD. Although direct evidence is lacking, antiplatelet or anticoagulant therapies have been shown to reduce D-dimer levels (29). Future studies could explore the value of combining anti-inflammatory and circulation-promoting therapies in elderly patients with AD.

Our data reveal a tight interplay between AD-driven systemic inflammation and a hypercoagulable state, underscoring the importance of age stratification when interpreting these biomarkers. However, this study was cross-sectional and drawn from a single center; therefore, we cannot draw causal conclusions, and whether these patterns hold in broader populations remains an open question. Prospective multicenter studies and mechanistic experiments are needed to confirm and extend our findings.

## 5 Conclusion

Our cohort of patients with AD displayed a triad of systemic inflammation, subclinical hypercoagulable state, and heightened allergic responses. Total IgE, SII, and PLR were reliable severity markers that performed reliably regardless of age. By contrast, coagulation markers proved age-dependent in their diagnostic value: DFR helped flag early disease progression in patients <60 years (AUC = 0.723), while D-dimer helped predict moderate-to-severe disease in those aged ≥60 years (AUC = 0.805). Based on these patterns, we suggest a two-tier, age-stratified approach to clinical evaluation: a core panel of total IgE, SII, and PLR for all patients with AD, supplemented with DFR in the <60 group and by D-dimer plus absolute eosinophil count in older patients. A word of caution: these findings come from an adult AD population (ages 12–80 years). They offer fresh perspectives on managing elderly patients with AD (≥60 years), but should not be directly applied to children or prepubertal cohorts.

## Data Availability

The original contributions presented in this study are included in the article/Supplementary Material, further inquiries can be directed to the corresponding authors.
